# 

**Published:** 1856-01

**Authors:** 


					THE
AMERICAN
JOURNAL
DENTAL
SCIENCE.
EDITED BY
CHAPIN A. HARRIS, M, D? D. D. S.
A. SNOWDEN PIGGOT, M. D.
"vol. 6?nsr e w series.
PHILADELPHIA
LINDSAY & BLAKISTON.
LONDON:
TRUBNER & CO. 12 PATERNOSTER ROW.
1856.
vj
1
AM 45 M

				

